# The roles of selectivity filters in determining aluminum transport by AtNIP1;2

**DOI:** 10.1080/15592324.2021.1991686

**Published:** 2021-10-28

**Authors:** Yuqi Wang, Enzong Xiao, Guorong Wu, Qing Bai, Feng Xu, Xiyue Ji, Chune Li, Li Li, Jiping Liu

**Affiliations:** a Key Laboratory for Water Quality and Conservation of the Pearl River Delta, Ministry of Education, School of Environmental Science and Engineering, Guangzhou University, Guangzhou, China; b Robert W. Holley Center for Agriculture and Health, USDA-ARS, Cornell University, Ithaca, NY, USA; c Plant Breeding and Genetics Section, School of Integrative Plant Sciences, Cornell University, Ithaca, Ny, USA

**Keywords:** Aluminum tolerance, aquaporin, ar/R selectivity filter, nodulin 26-like intrinsic protein, NPA motifs

## Abstract

Aquaporins (AQPs) are channel proteins involved in transporting a variety of substrates. It has been proposed that the constriction regions in the central pores of the AQP channels play a crucial role in determining transport substrates and activities of AQPs. Our previous results suggest that AtNIP1;2, a member of the AQP superfamily in Arabidopsis, facilitates aluminum transport across the plasma membrane. However, the functions of the constriction regions in AtNIP1;2-mediated transport activities are unclear. This study reports that residue substitutions of the constriction regions affect AtNIP1;2-mediated aluminum uptake, demonstrating the critical roles of the constriction regions for transport activities. Furthermore, a constriction region that partially or wholly mimics AtNIP5;1, a demonstrated boric-acid transporter, could not render the boric-acid transport activity to AtNIP1;2. Therefore, besides the constriction regions, other structural features are also involved in determining the nature of AtNIP1;2’s transport activities.

**Abbreviations**: AIAR: alanine-isoleucine-alanine-arginine; AIGR: alanine-isoleucine-glycine- arginine; AQP: aquaporin; Al-Mal: aluminum-malate; ar/R: aromatic/arginine; AVAR: alanine-valine-alanine-arginine; CK: control; H: helical domain; ICP-MS: inductively coupled plasma mass spectrometry; LA - LE: inter-helical loops A to E; NIP: nodulin 26-like intrinsic protein; NPA: asparagine-proline-alanine; NPG: asparagine-proline- glycine; NPS: asparagine-proline-Serine; NPV: asparagine-proline-valine; ORF: open reading frame; PIP: plasma membrane intrinsic proteins; SIP: small basic intrinsic proteins; TM: transmembrane helices; WIAR: tryptophan-isoleucine-alanine-arginine; WVAR: tryptophan-valine-alanine-arginine; WVGR: tryptophan-valine-glycine- arginine.

## Introduction

Aquaporins (AQPs) are channel proteins generally believed to facilitate the permeation of water and small uncharged solutes across the plasma and intracellular membranes.^1^ However, increasing evidence implicates that some AQP members potentially transport ions in plants.^2^

Aquaporins exhibit highly conserved structural features.^3^ Firstly, four AQP monomers form a biologically active tetramer embedded in cell membranes.^4, 5^ Secondly, each monomer contains an active pore region surrounded by six transmembrane helical domains (H1-H6) connected by five inter-helical loops, i.e., loop A to loop E (LA-LE).^5^ Thirdly, two major constrictions in the pore are thought to play critical roles in the functional specialization of AQPs.^6^

The pore’s first constriction comprises two highly conserved asparagine-proline-alanine (NPA) motifs in the hydrophobic LB and LE.^6–8^ Structurally, the asparagine (N) residues in the two NPA motifs fold back into the core of the protein to form one of the significant constrictions.^3,7,9^ The second constriction is located near the extracellular end of the pore, designated as an ar/R (aromatic/arginine) region due to the high prevalence of aromatic and basic residues.^7^ The ar/R region is comprised of four residues, one each from the helix 2 (H2) and helix 5 (H5) and two from the loop E (LE1 and LE2).^7,10,11^

It has been postulated that the NPA motifs function as a primary filter against protons and other positive ions.^6,7,12,13^ Structural and functional studies indicated that the NPA motif’s polar asparagine (N) residues are involved in hydrogen-bonding interactions with transport substrates.^7,14^ For instance, the hydrogen-bonding interactions between the N residues of the NPA motifs and water molecules are essential for maintaining the connectivity of water flow in the pore of the AQP-1 water channel.^14^ Hence, replacing the N residue with hydrophobic residues caused completely broken aqueous pathways of the AQP-1 channel.^14^

In contrast, the ar/R constriction is proposed as the primary filter for substrate selectivity.^8,10,11,15^ Furthermore, the highly conserved Arg (R) residue at the H2 position forms a hydrophilic surface and facilitates hydrogen bonding with the transport substrates.^8,16^ Thus, the R residue of the ar/R tetrad is critical for substrate selectivity in some AQP members.^17,18^ For instance, an exchange of the R residue altered substrate selectivity for some TIP members.^19^

Based on their sequence similarity, plant AQPs can be classified into four subfamilies: plasma membrane intrinsic proteins (PIPs), tonoplast intrinsic proteins (TIPs), nodulin26-like intrinsic proteins (NIPs), and small basic intrinsic proteins (SIPs).^20^ The NIP subfamily is unique to plants, and its members facilitate transporting a diversity of substrates, including water, glycerol,^21,22^ lactic acid,^23^ urea,^24^ formamide,^24^ silicic acid,^25^ selenite,^26^ aluminum,^27^ and metalloids such as arsenite (As(III)), antimonite (Sb(III)), and boron (B).^28–33^

Arabidopsis has nine NIP members that can be further divided into two subgroups, i.e., NIP-I and NIP-II, based on their different ar/R tetrad patterns.^8,9,34^ The NIP-I subgroup has six members, i.e., NIP1;1, NIP1;2, NIP2;1, NIP3;1, NIP4;1, and NIP4;2, with a conserved tetrad pattern of Trp (W) at H2, Val/Ile (V/I) at H5, Ala (A) at LE1, and Arg (A) at LE2. Moreover, the NPA-I members have an invariant NPA triad for the NPA1 motif, but variant NPA, NPG, or NPV triads in the NPA2 motif.^34^

In contrast, three NIP-II members, i.e., NIP5;1, NIP6;1, and NIP7;1, have a similar tetrad pattern except that a smaller Ala (A) residue replaces the bulkier Trp (W) in the first residue of the ar/R tetrad, which results in a broader ar/R constriction that accommodates for transporting larger substrates.^8^ Furthermore, it has been functionally demonstrated that the NPA-I members could transport formamide and glycerol besides water. However, NPA-IIs show very low water permeability but can transport urea and metalloids.^24,35^

Our recent studies suggest that AtNIP1;2, an NPA-I member, mediates the permeation of aluminum (Al), possibly in the form of aluminum-malate (Al-Mal) complexes, across the plasma membrane (PM) in Arabidopsis.^27^ AtNIP1;2 facilitates Al removal from the cell wall into the cytosol in the root-tip region and subsequent root-to-shoot translocation, which are critical steps for an internal Al-resistance mechanism in plants.^27,36,37^ Furthermore, we have demonstrated that the Al-activated and AtALMT1-mediated malate release into the root cell wall^38–40^ is a prerequisite for the AtNIP1;2-mediated function and resistance in Arabidopsis.^41^ However, the roles of the NPA motifs and ar/R selectivity filter in AtNIP1;2’s functions are still unclear.

*AtNIP5;1* is the best-characterized NIP-II member that encodes a channel protein responsible for permeation of boric acid (B) into roots under B limitation.^33,42^ In this study, we tested the roles of the NPA and ar/R constriction regions in determining substrate selectivity and transport activity of AtNIP1;2. Through a site-directed mutagenesis approach, critical residues in the constriction regions of AtNIP1;2 were subject to chemical nature changes, e.g., polar to nonpolar, or conversions to AtNIP5;1-like patterns. Evaluation of substrate selectivity and transport activity suggested that the constriction regions play critical roles in AtNIP1;2-mediated Al selectivity and transport. Furthermore, AtNIP5;1-like NPA and ar/R constriction regions are insufficient to render B transport activities to AtNIP1;2.

## Materials and methods

### Site-directed mutagenesis

The open reading frame (ORF) of *AtNIP1;2* was amplified by primers NIP1;2-F, 5ʹ-CTACggatccAAAATGGCGGAGATCTCGGGAAA-3ʹ and NIP1;2-R, 5ʹ-ATCCgcggccgcACGAGAGCTACCGTTTCGCA-3ʹ (the underlined sequences are restriction enzyme sites for *Bam*H I and *Not* I, respectively). The PCR product was cut with restriction enzymes *Bam*H I and *Not* I and cloned into the yeast expression vector, *pYES2*, to create a *pYES2-AtNIP1;2* plasmid. *AtNIP1;2* mutants were generated by site-directed mutagenesis using the following synthetic oligonucleotide primers. The lower case letters in the primer sequences represent mismatched nucleotides that introduced single amino-acid substitutions in the translated AtNIP1;2 proteins.

For NPA modification of AtNIP1;2:

N111LF,5ʹ-CGGTGCTCATTTCctTCCGGCCGTCACAATCGC-3ʹ

N111LR,5ʹ-CGATTGTGACGGCCGGAagGAAATGAGCACCGG-3ʹ

A113SF,5ʹ-GCTCATTTCAATCCGtCCGTCACAATCGCATTCGC-3ʹ;

A113SR,5ʹ-GCGAATGCGATTGTGACGGaCGGATTGAAATGAGC-3ʹ;

N230LF,5ʹ- GGGAGCATCGATGctTCCAGGACGAAGTTTAGG-3ʹ

N230LR,5ʹ-CTAAACTTCGTCCTGGAagCATCGATGCTCCCG-3ʹ

G232VF,5ʹ-GCATCGATGAATCCAGtACGAAGTTTAGGACCTGC-3ʹ;

G232VR,5ʹ-GCAGGTCCTAAACTTCGTaCTGGATTCATCGATGC-3ʹ.

For ar/R modification of AtNIP1;2:

W91AF,5ʹ-CAGGGATCGCCATCGTTgcGGGACTTACCGTCATG-3ʹ;

W91A R, 5ʹ-CATGACGGTAAGTCCCgcAACGATGGCGATCCCTG-3ʹ

V218I F, 5ʹ-CAACAGTGCTACTTAACaTcATAATTGCCGGGCCG-3ʹ

V218I R, 5ʹ-CGGCCCGGCAATTATgAtGTTAAGTAGCACTGTTG-3ʹ

A227G F, 5ʹ-GGCCGGTATCGGGAGgATCGATGAATCCAGGAC-3ʹ

A227G R, 5ʹ-GTCCTGGATTCATCGATcCTCCCGATACCGGCC-3ʹ

R233G F, 5ʹ-ATCGATGAATCCAGGAgGAAGTTTAGGACCTGC-3ʹ

R233G R, 5ʹ- CAGGTCCTAAACTTCcTCCTGGATTCATCGATG-3ʹ

In brief, high-fidelity PCR was performed to amplify the *pYES2-AtNIP1;2* plasmid with *Pfu* DNA polymerase and primer pairs listed above. The resulting PCR products were checked by agarose gel electrophoresis. PCR products with the right size of 6.7 kb were digested with *Dpn*I at 37°C for 3 h, which cut the methylated *pYES2-AtNIP1;2* template into small fragments but left the non-methylated and circular PCR products intact. After *Dpn*I digestion, the PCR products were transformed into competent *E. coli* strain TOP10. The purified plasmids from the transformed TOP10 cells were verified by sequencing. Additional runs of PCR-based site-directed mutagenesis were performed to generate multiple mutations.

## Yeast Al sensitivity and uptake analysis

For Al sensitivity evaluation, the *pYES2* empty vector or *pYES2* carrying wild-type or mutant *AtNIP1;2* open reading frames (ORFs) were transformed into the yeast (*Sacchromyces cerevisea*) strain BY4741. The resultant lines were first cultured in a liquid SD-Ura medium to the stationary phase. Cells were collected by centrifuge at 5,000 g for 5 min, followed by wash 3 times with ddH_2_O and 3 times with a low pH, low magnesium (LPM) medium, buffered with 5 mM Succinic acid to pH 4.2. The LPM medium contained the following macronutrients in mM: (NH_4_)_2_SO_4_, 40; KCl, 5; NaCl, 2; CaCl_2_, 0.1; KH_2_P0_4_, 0.01; MgSO_4_, 0.25; the following micronutrients in μM: FeCl_3_,1; H_3_BO_3_,10; KI, 0.5; MnSO_4_, 2.5; Na_2_MoO_4_, 1; ZnSO_4_, 1.5; the following amino acids in mg/l: tyrosine, 0.03; glutamic acid, 0.075; adenine, 0.02; uracil, 0.02; phenylalanine, 0.05; valine, 0.15; serine, 0.4; leucine, 0.03; isoleucine, 0.03; lysine, 0.03; tryptophan, 0.02; arginine, 0.02; histidine, 0.02; methionine, 0.02; aspartic acid, 0.0625; threonine, 0.2; the following vitamins in ng/l: folic acid, 0.2; biotin, 0.2; *p*-aminobenzoic acid, 20; riboflavin, 20; calcium pantothenate, 40; niacin, 40; pyridoxine hydrochloride, 40; thiamine hydrochloride, 40; inositol, 200; and 2% galactose.

For the drop assay, 5 μl of 10-fold serially diluted cell suspensions were spotted onto solid LPM plates (pH to 4.2), containing 0, 100, or 200 μM Al-Mal (1:2) and 2% galactose for induction of the GAL promoter. The plates were photographed after incubation at 30°C for 4 days.

For determining Al and B contents, yeast cells were cultured in an LPM liquid medium (-Ura, +2% galactose and 1% raffinose, pH 4.2) to a mid-exponential phase. Cells were harvested by centrifuge at 5,000 g for 5 min, followed by 3 time washes with an LPM medium (-Ura, +2% galactose, 1% raffinose). After washing, the cells were transferred to a new LPM medium (2% galactose, pH 4.2 or 7.0 adjusted by 5 mM succinic acid) to an OD600 value at 3.0. Next, Al-malate or H_3_BO_3_ was added to the cell culture to a final concentration of 50 μM at pH 4.2 or 7.0, respectively. After 2 h incubation with gentle shaking, cells were harvested by centrifuge at 5000 × g for 5 min and washed 3 times with deionized water (ddH_2_O) (MilliQ; Millipore), dried, and then digested with 2 N HCl. The Al and B contents of each digested sample were determined by inductively coupled plasma mass spectrometry (ICP-MS) using an Agilent 7500 Series ICP mass spectrometer. Three biological replicates were conducted.

## Results and discussion

### Sequence comparison of the NPA and ar/R constriction regions between AtNIP1;2 and AtNIP5;1

**Figure 1.**
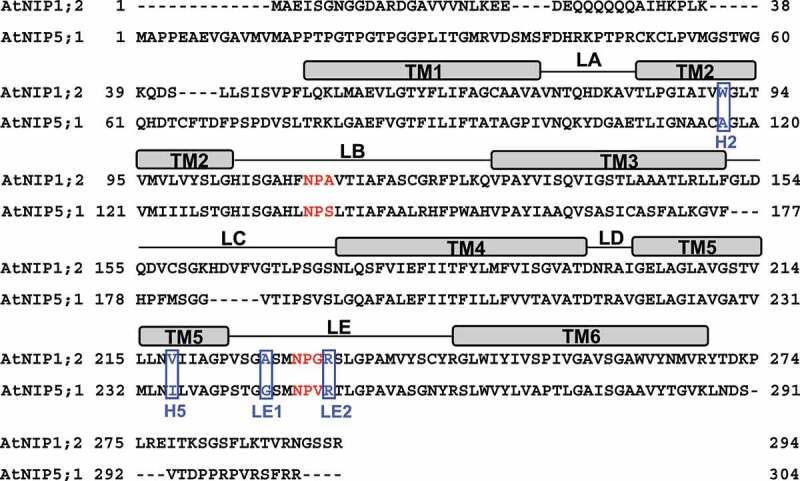
Sequence alignments of AtNIP1;2 and AtNIP5;1. Red letters define the NPA1 (in LB) and NPA2 (in LE) motifs. Blue boxed letters refer to the tetrad residues of the ar/R constriction region. Rectangle boxes above the amino-acid sequences delineate the transmembrane helices (TM). Thin lines between TMs indicate the inter-helical loops. H, helix; LA-LE, inter-helical loops A-E.

Sequencing alignment indicated that AtNIP1;2, a NIP-I member, has an NPA (Asn-Pro-Ala) and an NPG (Asn-Pro-Gly) sequence of NPA1 and NPA2 motifs, respectively. In contrast, the NIP-II member AtNIP5;1 has NPS (Asn-Pro-Ser) and NPV (Asn-Pro-Val) for the NPA1 and NPA2 motifs, respectively (Figure 1).^24^ Therefore, the third residue of both NPA motifs differs between AtNIP1;2 and AtNIP5;1 (Figure 1). Furthermore, AtNIP1;2 has a WVAR sequence of the ar/R region versus AIGR in AtNIP5;1. Thus, the ar/R tetrad sequences of AtNIP1;2 and AtNIP5;1 differ at the H2, H5, and LE1 positions (Figure 1).

## Influence of the NPA and ar/R constrictions on AtNIP1;2-mediated aluminum sensitivity in yeast

The asparagine (N) residue in the NPA1 and NPA2 motifs and the arginine (R) residue in the ar/R selective filter are conserved in the NIP subfamily.^34^ To investigate the role of these conserved residues in AtNIP1;2-mediated Al-uptake activities, we constructed three AtNIP1;2 single mutants, designated as N111L, N230L, and R233G. In these mutants, the polar N residue of NPA1 and NPA2 was replaced by a nonpolar leucine (L), while the R residue of the ar/R tetrad was replaced by glycine (G).

**Figure 2.**
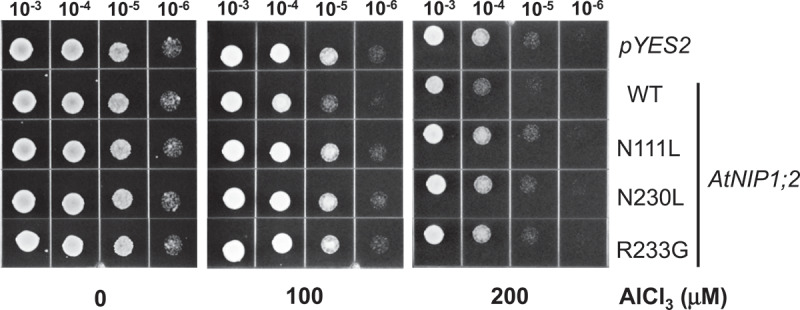
Mutations in the NPA motifs and ar/R selectivity filter eliminate AtNIP1;2- mediated aluminum sensitivity in yeast cells. Yeast (BY4741) lines carrying the empty vector *pYES2*, or constructs expressing the wild-type (WT) AtNIP1;2 (NPA/NPG/AIGR) or AtNIP1;2 mutants N111L (LPA/NPG/AIGR), N230L (NPA/LPG/AIGR), or R233G (NPA/NPG/AIGG) were subjected to Al sensitivity tests. Letters in parenthesis refer to the NPA1, NPA2, and ar/R constriction regions, respectively. Red letters indicate exchanged residues. Aliquots (5 μl) of 10-fold serial dilutions of re-suspended cells were spotted onto LPM plates (pH 4.2, 2% galactose) supplemented without (no stress) or with 100 and 200 μM AlCl3 buffered with 200 and 400 μM malate, respectively. The LPM plates were placed in a 30°C incubator for 3 d.

Aluminum uptake mediated by the wild-type and mutant AtNIP1;2 proteins were investigated by assessing yeast [*S. cerevisiae* (BY4741)] sensitivity to Al toxicity. Yeast lines harboring an empty *pYES2* vector (control, CK) or expressing *AtNIP1;2* and its mutants were subjected to Al treatment on low pH, low magnesium (LPM) agar plates (pH 4.2) (Figure 2).

The growth of yeast cells transformed with the empty *pYES2* vector or *pYES2* containing the wild-type or mutant *AtNIP1;2* could not be distinguished on the LPM plates that were not supplemented with Al (Figure 2). This result indicated that heterologous expression of AtNIP1;2 or its mutants has no harmful effect on yeast growth under standard conditions.

However, yeast cells that expressed wild-type *AtNIP1;2* showed significantly reduced growth compared to those carrying the empty vector when exposed to Al stresses by adding 100 or 200 μM Al-malate (Al-Mal) to the growth medium (Figure 2). The growth inhibition was presumed to be due to AtNIP1;2-mediated Al uptake and accumulation, as Wang et al. (2017) reported previously.

In contrast, N111L, N230L, and R233G grew similarly to the empty vector control (CK) line under – and + Al treatments (Figure 2). These results indicated mutations in the conserved N and R residues on the NPA motifs and the ar/R region, respectively, abolished AtNIP1;2-mediated Al sensitivity in yeast (Figure 2), suggesting critical roles of these residues in AtNIP1;2-mediated Al-Mal uptake and accumulation.

## Importance of the NPA and ar/R constriction regions for AtNIP1;2-mediated Al uptake

**Figure 3.**
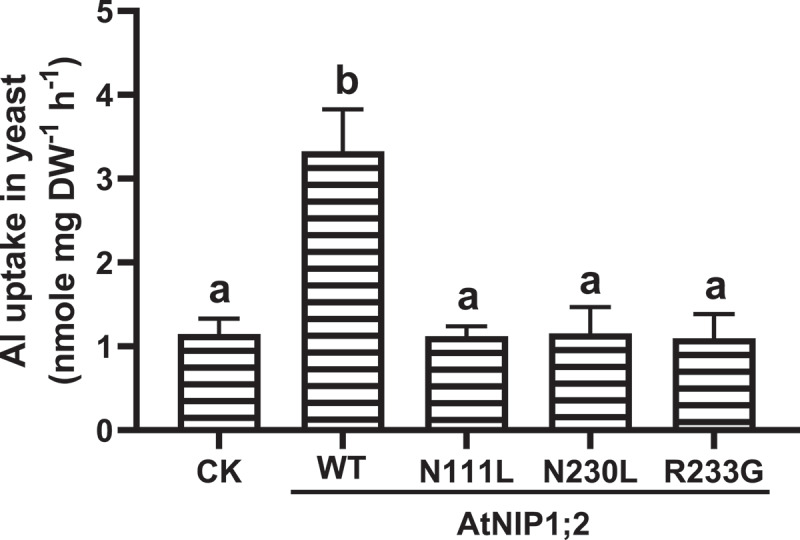
Influence of N/L substitution in the NPA motifs and R/G substitution in the ar/R selectivity filter on AtNIP1;2-mediated Al uptake. Yeast (BY4741) lines carrying the empty control (CK) vector *pYES2* and expressing the native or mutated (i.e., N111L, N230L, and R233G) AtNIP1;2 were subject to short-term (2 h) Al uptake assays. Data are means ± SD (n = 3). Different letters above the columns indicate statistically significant differences at *P* < .05 by Tukey’s test.

To confirm the differences in Al sensitivity were associated with Al uptake and accumulation in the yeast cells, short-term (2 h) Al uptake was evaluated for individual yeast lines. As indicated in Figure 3, the yeast line harboring the native AtNIP1;2 construct had an ~3-fold higher Al uptake rate than the control (CK) line. This result is consistent with our previous observation that AtNIP1;2 facilitates across-PM Al uptake in yeast.^27^

In contrast, the Al uptake in the yeast lines expressing AtNIP1;2 mutants (N111L, N230L, or R233G) was comparable to those of the CK line but not the native AtNIP1;2 line (Figure 3), indicating that that the mutations caused a loss of AtNIP1;2-mediated Al-Mal uptake in yeast. Thus, the asparagine (N) residue in the NPA motifs and the arginine (R) residue of the ar/R selective filter are critical for AtNIP1;2-mediated across-membrane Al uptake.

## Influence of the NPA and ar/R constrictions on substrate selectivity of AtNIP1;2

Except for the conserved 1^st^ residues of N in NPA1 and NPA2 motifs and the 4^th^ residue of R in the ar/R region, other residues show more diversity between the NIP-I and NIP-II subgroups.^34^ For instance, AtNIP1;2, a NIP-I member, and AtNIP5;1, a NIP-II member, differ in the 3^rd^ residues of the NPA motifs and the first three residues of the ar/R tetrad (Figure 1).

To investigate the impact of the identity of pore constriction regions on uptake activities and substrate selectivity, the NPA motifs and ar/R selective filter of AtNIP1;2 were exchanged to partially or entirely mimic those in AtNIP5;1, a demonstrated B transporter of the NIP-II subgroup.^33^

**Figure 4.**
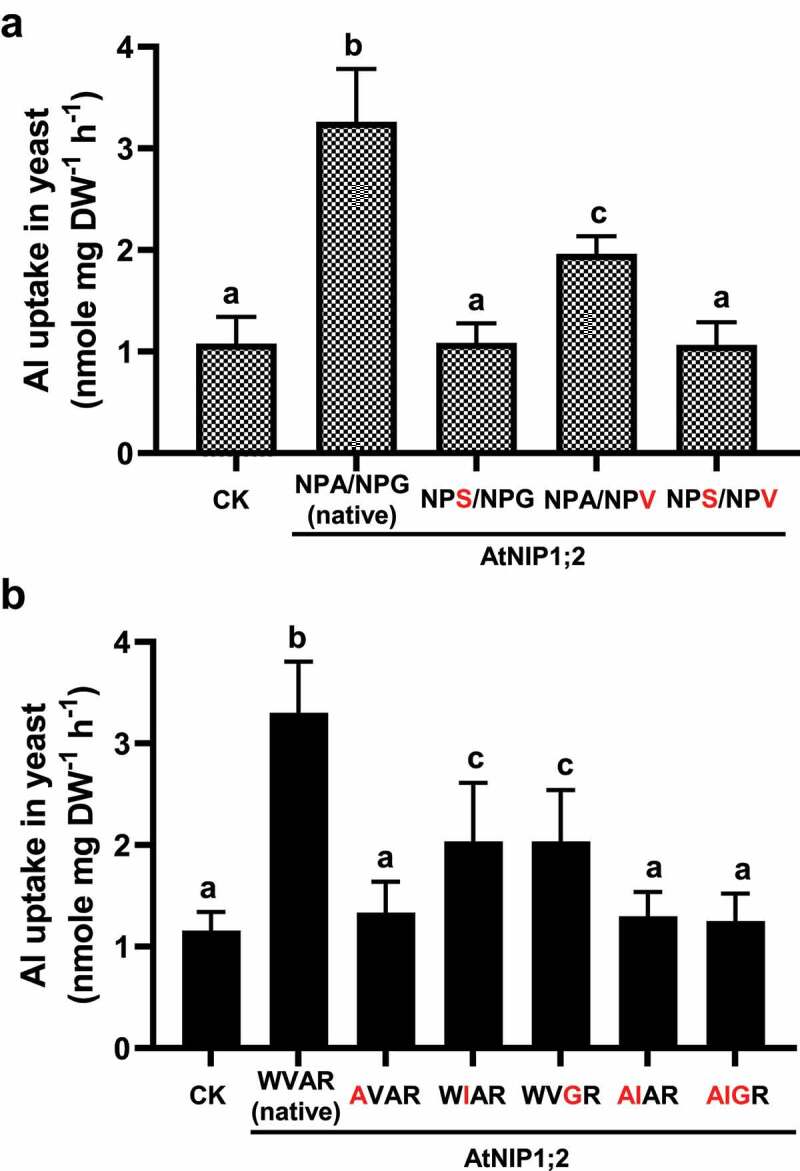
Impacts of third residue substitutions in the NPA motifs and residue substitutions in the ar/R selectivity filter on AtNIP1;2-mediated Al uptake. Yeast (BY4741) lines carrying the control (CK) empty vector *pYES2* and the native (NPA/NPG/WVAR) or mutated AtNIP1;2 were subject to short-term (2 h) Al-uptake assays. (a) Red letters indicate substitutions of the third residue in the NPA1 and NPA2 motifs. (b) Red letters indicate residue substitutions of the ar/R tetrad. Data are means ± SD (n = 3). Different letters above the columns indicate statistically significant differences among groups at *P* < .05 by Tukey’s test.

As indicated in Figure 4a, a single alanine-to-serine (A113S) exchange at the 3^rd^ residue of the NPA1 motif (NPA to NPS) abolished the AtNIP1;2-mediated Al-Mal transport activity (Figure 4a). This result indicates that the third residue of S is as critical as the first residue of N in the NPA1 motif for the AtNIP1;2-facilitated Al uptake (Figure 3). In contrast, a glycine-to-valine (G232V) exchange at the 3^rd^ residue of the NPA2 motif had a much weaker impact on Al uptake: Al uptake activity decreased ~40% compared with the native AtNIP1;2 (Figure 4a). Furthermore, the double AtNIP1;2 mutant (A113S/G232V), carrying NPA1/NPA2 motifs mimicked to those of AtNIP5;1 (NPS/NPV), also showed no increased Al transport activities (Figure 4a). This result indicates that the effect of A113S overrides that of G232V. Similarly, a single AVAR mutant, which contained a tryptophan-to-alanine (W91A) substitution that mimicks the first residue of the ar/R tetrad in AtNIP5;1, did not show any Al-uptake activity compared with the control line (Figure 4b).

The strong effects of the A-to-S in NPA1 and W-to-A in the ar/R tetrad on AtNIP1;2-mediated Al transport could be because of the serine’s hydroxyl group and tryptophan indole group affecting the folding and orientation of the channel protein. Or, the A-to-S substitution at NPA1 may affect post-translational modification, e.g., phosphorylation.^43,44^

Aluminum-uptake phenotypes differed in the other two single mutant lines, i.e., WIAR and WVGR (Figure 4b). The WIAR and WVGR lines contained valine-to-isoleucine (V218I) and alanine-to-glycine (A227G) exchanges that mimicked the 2^nd^ and 3^rd^ residues of the ar/R tetrad in AtNIP5;1. As shown in Figure 4b, Al uptake decreased ~38% in the WIAR and WVGR mutant lines than the wild-type (native) AtNIP1;2 (Figure 4b). These results suggest that the 2^nd^ and 3^rd^ residues have fewer impacts on the AtNIP1;2-mediated Al uptake than the 1^st^ (Figure 4b) and 4^th^ (Figure 3) residues of the ar/R tetrad.

Furthermore, a double AIAR mutant and a triple AIGR mutant contained W91A/V218I and W91A/V218I/A227G residue substitutions partially and wholly mimicked the AtNIP5;1 ar/R tetrad. Both AIAR and AIGR lines resembled the AVAR single mutant in Al-uptake activities (Figure 4b). This result indicates that the tryptophan (W) residue at H2 has a dominant effect on influencing AtNIP1;2-mediated Al uptake over the valine (V) and alanine (A) residues at H5 and LE1, respectively.

## AtNIP5;1-like constriction regions could not render boron transport activity to AtNIP1;2

**Figure 5.**
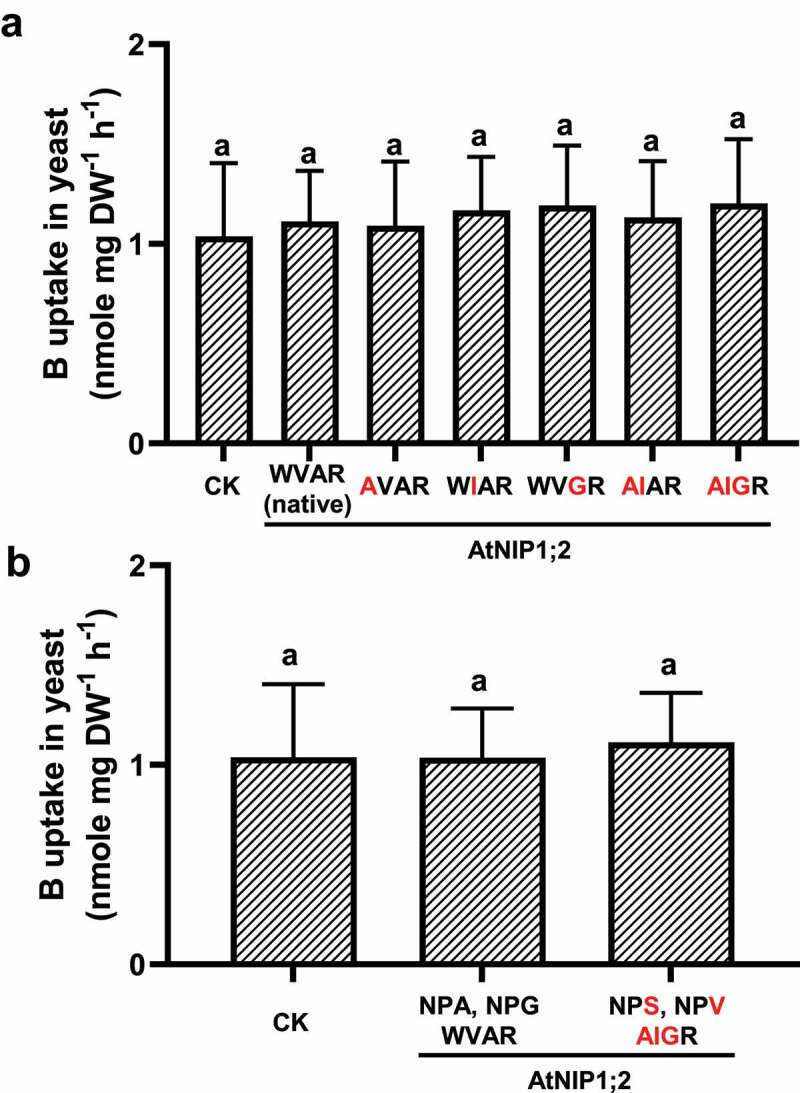
AtNIP5;1-like NPA motifs and ar/R selectivity filter does not render B uptake activity to AtNIP1;2. Yeast (BY4741) lines carrying the control (CK) empty vector *pYES2* or expressing AtNIP1;2 containing the native or mutated NPA and ar/R constriction regions were subject to short-term (2 h) B uptake assays. (a) Red letters indicate residue substitutions in the ar/R tetrad. (b) Residue substitutions (red letters) that transform the AtNIP1;2 NPA and ar/R constriction regions to an AtNIP5;1 type. Data are means ± SD (n = 3). Different letters above the columns indicate statistically significant differences at *P* < .05 by Tukey’s test.

To test whether an ar/R tetrad that mimics AtNIP5;1 can render B transport activity to AtNIP1;2, tryptophan (W) at H2, valine (V) at H5, or alanine (A) at LE1 in AtNIP1;2 were changed individually or in combination to alanine (A), isoleucine (I), or glycine (G), respectively (Figure 5a). No B uptake activities were observed for the native AtNIP1;2 and its single, double, or triple ar/R mutants (Figure 5a).

We then generated a quintuple mutant (NPS/NPV/AIGR, the underlines indicate residue substitutions) where the NPA motifs and ar/R selective filter mimics AtNIP5;1. However, as shown in Figure 5b, this AtNIP1;2 quintuple mutant could not transport B also.

These results indicate that 1) boric acid is not a transport substrate for AtNIP1;2 as the native AtNIP1;2 is incapable of transporting boric acid; 2) partial or complete mimics of the NPA motifs and the ar/R tetrad of AtNIP5;1 could not convert AtNIP1;2 from an Al to a B transporter.

In conclusion, the substrate selectivity of AtNIP1;2 is not simply controlled by the NPA motifs and ar/R selectivity filter. Other structural features and post-translational modifications, e.g., phosphorylation,^43,44^ methylation and acetylation,^45^ and glycosylation,^46^ may also be necessary for determining the nature of the substrate specificity and transport activities.

## Data Availability

The data supporting this study’s findings are available from the corresponding author, JL, upon reasonable request.
